# Reoperation After Cholecystectomy. The Role of the Cystic Duct Stump

**DOI:** 10.1155/1991/57017

**Published:** 1991

**Authors:** M. A. Rogy, R. Függer, F. Herbst, F. Schulz

**Affiliations:** Department of Surgery I University of Vienna Medical School Alserstr. 4 Vienna A-1090 Austria

## Abstract

The so-called “Postcholecystectomy Syndrome” may be due to various pathological biliary causes. The aim of this study was to evaluate the significance of the cystic duct stump syndrome and if so, how often a long (>1.5 cm) cystic duct stump was an indication for reoperation on the bile ducts after cholecystectomy in our patients. Three hundred and twenty two patients underwent a second operation on the bile ducts after cholecystectomy in the last ten years. In 35 patients (10.8%) a striking finding was a long cystic duct stump (>1.5 cm). In 24 of these patients, a pathological finding, in addition to the long cystic duct stump, was found on exploration. Out of these 24 patients there were 14 with common bile duct stones; 6 with stenosis of the sphincter of Oddi; 3 with chronic pancreatitis and in one patient hepatitis was the cause of the symptoms. From the remaining 11 patients 8 had a stone in a partial gall bladder or cystic duct stump. One patient had a fistula between the cystic duct stump and duodenum and one a suture granuloma. There was only one patient where a 1.5 cm long cystic duct stump remnant was the only pathological finding. Four years after reoperation this patient is still suffering from the same intermittent gastrointestinal symptoms. We conclude that the cystic duct stump is hardly ever a cause for recurrent symptoms in itself. Total excision of the cystic duct does not eliminate the existence of postcholecystectomy symptoms.


HPB Surgery, 1991, Vol. 4, pp. 129–135
Reprints available directly from the publisher
Photocopying permitted by license only

© 1991 Harwood Academic Publishers GmbH
Printed in the United Kingdom

### REOPERATION AFTER CHOLECYSTECTOMY. THE ROLE OF THE CYSTIC DUCT STUMP

M.A. ROGY, R. FÜGGER, F. HERBST, and F. SCHULZ
*Department of Surgery I, University of Vienna Medical School, Austria*

*(Received 18 January 1991)*

The so-called "Postcholecystectomy Syndrome" may be due to various pathological biliary causes. The aim of this study was to evaluate the significance of the cystic duct stump syndrome and if so, how often a long (>1.5 cm) cystic duct stump was an indication for reoperation on the bile ducts after cholecystectomy in our patients. Three hundred and twenty two patients underwent a second operation on the bile ducts after cholecystectomy in the last ten years. In 35 patients (10.8%) a striking finding was a long cystic duct stump (>1.5 cm). In 24 of these patients, a pathological finding, in addition to the long cystic duct stump, was found on exploration. Out of these 24 patients there were 14 with common bile duct stones; 6 with stenosis of the sphincter of Oddi; 3 with chronic pancreatitis and in one patient hepatitis was the cause of the symptoms. From the remaining 11 patients 8 had a stone in a partial gall bladder or cystic duct stump. One patient had a fistula between the cystic duct stump and duodenum and one a suture granuloma. There was only one patient where a 1.5 cm long cystic duct stump remnant was the only pathological finding. Four years after reoperation this patient is still suffering from the same intermittent gastrointestinal symptoms. We conclude that the cystic duct stump is hardly ever a cause for recurrent symptoms in itself. Total excision of the cystic duct does not eliminate the existence of postcholecystectomy symptoms.

KEY WORDS: Cystic duct stump, reoperation after cholecystectomy, postcholecystectomy complaints

### INTRODUCTION

Overall, cholecystectomy is an established successful operation which provides total relief of presurgical symptoms in up to 90% of patients. The incidence of gastrointestinal symptoms after cholecystectomy has been reported to be between 10% and 50%[1,2,3]. Fortunately, these complaints are usually mild and nonspecific and consist mainly of transient nausea, indigestion, belching, bloating and flatulence.

However, about 5% of patients after cholecystectomy experience severe episodes of upper abdominal pain similar to those that they had prior to cholecystectomy[4,5,6]. The most common cause of persistent postcholecystectomy symptoms is an overlooked extrabiliary disorder (e.g. reflux esophagitis, peptic ulceration, chronic pancreatitis)[7,8,9,10,11].

In a small percentage of patients, however, a disorder of the extrahepatic bileducts may result in persistent symptoms. These so called postcholecystectomy syndromes may be due to (1) biliary strictures, (2) retained biliary calculi, (3) cystic

Address correspondence to: M.A. Rogy, M.D., Dept. of Surgery I, A-1090 Vienna, Alserstr. 4, Austria.

duct stump syndrome (4) stenosis or dyskinesia of the sphincter of Oddi, or (5) bile salt-induced diarrhea or gastritis.

The purpose of this study was to evaluate whether there is a cystic duct stump syndrome and if so, how often a long cystic duct stump was the indication for reoperation on the bile ducts after cholecystectomy in our patients.

### MATERIALS AND METHODS

Three thousand six hundred and eighty nine patients were operated on for benign disease of the extrahepatic biliary tract between January 1979 and January 1989. Most of these operations were ordinary cholecystectomies. Three hundred and twenty two patients underwent a second operation on the bile ducts after cholecystectomy (Table 1).

**Table 1:**
**Indication for reoperation after cholecystectomy in 322 patients**

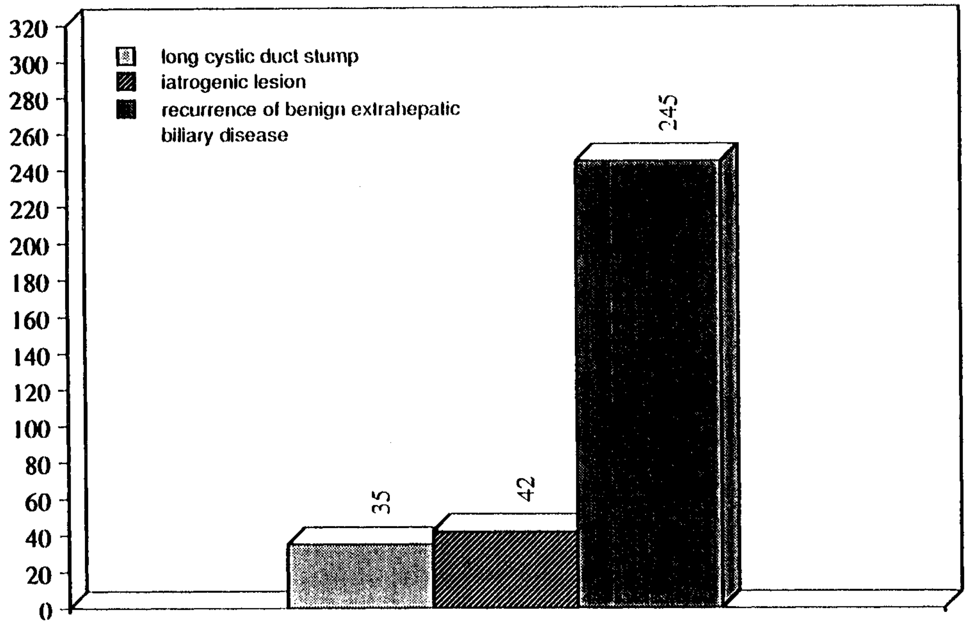

In most of these cases, the primary operation, cholecystectomy, was performed in another hospital. Out of the three hundred and twenty two patients there were two hundred and twenty seven women and ninety five men with a mean age of 61 years. All patients experienced severe episodes of upper abdominal pain, similar to those they had prior to cholecystectomy. The time between the primary operation and the reoperation was between 1 year and 44 years with a median of 9 years interval. The records and the operation reports of these patients were investigated retrospectively. In 35 patients (28 male, 7 female) mean age 61 years, who are the subject of our study, a striking finding was a long cystic duct stump (>1.5 cm) described in the operation report, which led to further investigation.

### RESULTS

The cause of complaints in 23 patients was a pathological finding within the bile duct system (14 common bile duct stones, 6 stenosis of the sphincter of Oddi), and chronic pancreatitis in 3 patients. The long cystic duct stump was just an associated finding. Hepatitis was the cause of complaints in one patient (Table 2).

A pathological finding of the cystic duct stump was causative for complaints in 11 further patients (7 partial gall bladder with stones, 1 stone within the cystic duct stump, 1 fistula between cystic duct stump and duodenum, 1 suture granuloma) (Table 3). There was only one patient where a one and a half cm long cystic duct stump was the only pathological finding. Four years after the reoperation this patient is still suffering from the same intermittent gastrointestinal symptoms, namely upper abdominal pain in connection with postprandial bloating and belching.

**Table 2** Reasons for postcholecystectomy complaints other than the cystic duct stump remnant (with associated long cystic duct stump).

| | |
|---|---|
| Common bile duct stones | 14 pat. |
| Stenosis of the sphincter of Oddi | 6 pat. |
| Chronic pancreatitis | 3 pat. |
| Hepatitis | 1 pat. |

**Table 3** Reasons for postcholecystectomy complaints with associated long cystic duct stump.

| | |
|---|---|
| Partial gallbladder with stones | 7 pat. |
| Stones within the cystic duct stump | 1 pat. |
| Fistula between the cystic duct stump and duodenum | 1 pat. |
| Suture granuloma at the cystic duct stump | 1 pat. |
| Long cystic duct stump | 1 pat. |

### DISCUSSION

Symptoms resembling biliary colic or cholecystitis in the postcholecystectomy patient have frequently been attributed to disease in a long (>1.5 cm) cystic duct remnant (cystic duct stump syndrome). There are various papers[12,13,14,15,16] reporting that a cystic duct remnant can cause symptoms even after the common duct calculi had been removed. These investigators also reported examples of the

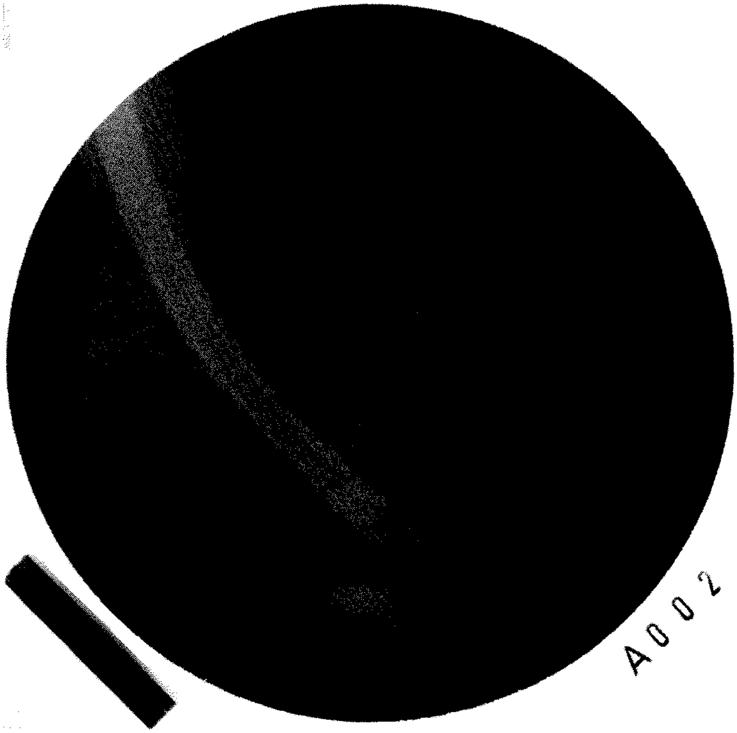

**Figure 1** The cystic duct stump remnant illustrated by ERC.

presence of calculi in both the cystic duct remnant and the common duct and expressed the view that calculi could be formed in the cystic duct remnant. Pain in the right upper quadrant, sometimes with radiation to the right shoulder was found to be the outstanding symptom and jaundice its commonest sign. Careful analysis, however, reveals that postcholecystectomy complaints are attributable to other causes in almost all patients in whom the symptom complex was originally thought to result from the existence of a long cystic duct stump[17,18]. Glenn and McSherry[19] examined the question of postcholecystectomy problems by analysing the reasons for reoperation on two hundred and fifty three patients who had previously undergone a cholecystectomy. They did not figure out the role of the cystic duct stump particularly, but the results were similar to ours. The majority of the patients were found to have disease of the bile ducts, liver or pancreas.

Barnett *et al.*[20] reported a case of an intraluminal bile duct filling defect caused by an inverted cystic duct stump remnant. They found this condition during ERC for choledocholithiasis. Intraluminal bile duct filling defects are typically due to neoplasm or retained stones. Primary biliary carcinomas are rare, and benign neoplasms of the extrahepatic bile ducts are even more unusual[21].

Nelson[22] observed two cases with a cystic duct stump fistula as in our patient. A case of malignant papilloma of a cystic duct stump which developed within the lumen and was the cause of clinical episodes of pain, jaundice, fever and gastrointestinal hemorrhage is reported by Ferdinando Carotenuto *et al*[23]. So, there are many who report on the cystic duct stump remnant as an etiologic factor for distress after cholecystectomy, justifying reoperation and excision of the stump.

However, a careful search through the literature shows that almost all of these reports are case reports. In fact these reports show the cystic duct stump remnant involved with various pathological findings of the extrahepatic biliary tract but the cystic duct remnant in itself is hardly ever a cause for recurrent symptoms.

The duct is quite well visualized by ERC[24,25], which seems until now to be the first choice for investigation of the extrahepatic biliary tract, followed by various other techniques like CT, biliary tract radionuclide studies and percutaneous transhepatic cholangiography. Intravenous cholangiography, due to anaphylactic reactions to the contrast medium plays a much less important role. In our own series ERC (Figure 1) has replaced the i.v. cholangiogram in recent years. As already pointed out in the literature our data confirm that a cholangiographic finding of a cystic duct stump alone does not justify surgical intervention, since in a number of patients troubles derive from elsewhere in the biliary tract or from adjacent organs. This is also very important for the newer techniques of endoscopic cholecystectomy where it is not possible to explore and shorten the cystic duct as you should do in simple cholecystectomy.

In conclusion our results show that the role of the long cystic duct stump remnant as a reason for reoperation after cholecystectomy is negligible. If cholangiography reveals the presence of a long cystic duct stump and where there is no associated secondary pathological finding involving the cystic duct, the stump is not responsible for any organic post surgical symptoms.

### *References*

1. Bodvall, B. (1973) The postcholecystectomy syndromes. *Clin. Gastroenterol.*, **2**(1), 103–125
2. Christiansen, J. and Schmidt, A. (1971) The postcholecystectomy syndrome. *Acta Chir. Scand.*, **137**, 789–793
3. Ekdahl, P.H. (1953) On late distress following biliary tract operations. *Acta Chir.Scand.*, **106**, 339
4. Bar-Meir, S., Halpern, Z. and Bardan, E. *et al.* (1984) Frequency of papillary dysfunction among cholecystectomized patients. *Hepatology*, **4**(2), 328–330
5. Stefanini, P. and De Barnardinis, G. (1974) Factors influencing the long term results of cholecystectomy. *Surg. Gynecol. Obstet.*, **139**, 735–738
6. Glenn, F. and Cameron, J.L. (1981) Complications following operations upon the biliary tract and their management. In: Hardy J.D., ed. *Complications in surgery and their management*, pp. 512–518. Philadelphia: WB Saunders
7. Maingot, R. (1974) Postoperative stricture of the bile ducts causes and prevention: diagnosis: reconstruction operations, abdominal operations, 6th ed, Vol 1, pp. 1124–1176. New York: Appleton Century Croft
8. Adam, Y.G., Rosen, A., and Oand, J. *et al.* (1983) Giant bile cyst following cholecystectomy. *J.Clin. Gastroenterol.* **5**, 267–269
9. Rath, J.L.A., (1985) Postcholecystectomy syndrome. In: Berk J.E., ed. *Bakus Gastroenterology*, 4th ed., pp. 3815–3833. Philadelphia: W.B. Saunders
10. Greenstein, A.J. and Dreiling D.A. (1973) The normal intravenous cholangiogram following cholecystitis: a clue to the cystic duct stump syndrome. *Am. J. Gastroenterol.*, **59** (2), 134–140
11. Larson, D.M. and Storsteen, K.A. (1984) Traumatic neuroma of the bile ducts with intrahepatic extension causing obstructive jaundice. *Hum. Pathol.*, **15** (3), 287–289
12. Daniels, V., Schmiedt, H.D., Lenner, V. and Bruenner, H. (1980) Langer Zystikusstumpf als Ursache der Restbeschwerden nach Cholezystektomie. *Leber Magen Darm*, **10** (4), 207–212
13. Koele, W. and Mueller, V. (1979) Sogenanntes "Zystikusstumpfsyndrom" -- eine kritische Analyse. *Zentralbl. Chir.*, **104** (9), 551–556

14. Lueders, H., Wandt, U. and Werner, G. (1988) Morphological findings at the cystic duct stump a study to determine the frequency of cystic stump neuromas after cholecystectomy. *Z. Klin. Med.*, **43**, 1537--1539
15. Parmeggiani, A. and Alemanno, R. (1986) The cystic stump syndrome clinical case histories. *Acta Chir. Ital.*, **41**(5), 652–657
16. Berger, H., Weinzierl, M., Neville,Es, and Pratschke, E. (1989) Percutaneous transcatheter occlusion of cystic duct stump in postcholecystectomy bile leakage. *Gastrointest. Radiol.*, **14**, 334–336
17. Tritapepe, R., Pozzi, C., Montorsi, M. and Doldi, S.B. (1989) The cystic duct stump syndrome reality or fantasy. *Ann., Ital. Chir.*, **60**(3), 133–136
18. Aarimaa, M. and Makela, P. (1981) The cystic duct stump and the postcholecystectomy sysndrome. An analysis of 54 patients subjected to ERCP. *Annales Chirurgiae et Gynaecologiae,* **70**(6), 297–303
19. Glenn, F. and McSherry, C.K. (1965) Secondary abdominal operations for symptoms following biliary tract surgery. *Surg. Gynecol. Obstet.*, **121**, 979–988
20. Barnett, J.L., Scheimann, J.M. and Grace, H.E. (1988) The cystic duct remnant: An unusual case of a biliary intraluminal filling defect. *Am.J.Gastroenterol.*, **83**(10), 1189–1191
21. Orloff, M.J. and Marassi, N.P. (1985) Tumors of the extrahepatic bile ducts. In: Berk, J.E., ed. *Bakus Gastroenterology,* 4th ed., pp. 3771–3781. Philadelphia: W. B. Saunders
22. Nelson, A.M. (1984) Cystic duct fistula: A complication of cholecystectomy. *The American Journal of Gastroenterology* **79**(6), 479–481
23. Carotenuto, F. and Simi, M. (1974) Carcinomatous papilloma of the cystic stump (report of a case). *Surgery in Italy,* **3/4**, 253–256
24. Weissmann, H.S., Frank, M. and Rosenblatt, R. *et al.* (1979) Cholescintigraphy and ultrasonography and computed tomography in evaluation of biliary tract disorders. *Semin. Nucl. Med.*, **9**, 22–29
25. Janardhanan, R., Brodmerkel, G.J., Turowski, P., Gregory, D.H. and Agrawal, R.M. (1986) Endoscopic retrograde cholangiopancreatography cystic duct leaks. *The American Journal of Gastroenterology*, **81** (6), 474–476

*(Accepted by S. Bengmark on 18 January 1991)*

### INVITED COMMENTARY

Rogy and his colleagues have reviewed the results of reoperation after cholecystectomy in 322 patients and found 35 patients with long cystic duct remnants. In 24 of these patients preoperative symptoms could be explained by pathologic findings in addition to the long cystic duct. In 10 patients pathology within the cystic duct remnant such as stones (eight), a fistula to the duodenum (one) or a suture granuloma (one) caused preoperative pain. The one patient without additional pathology continued to have symptoms after excision of the cystic duct remnant. The authors conclude that the cystic duct stump itself rarely causes symptoms and that excision of a cystic duct remnant without associated pathology will not eliminate postcholecystectomy symptoms. This report and other recent analyses support these conclusions.

The authors have defined a "long' cystic duct remnant as those greater than 1.5 cm. They found that approximately 10% of their patients had cystic duct stumps that were longer than 1.5 cm. Some authorities argue, however, that 1.5 to 2.0 cm is an appropriate length for the cystic duct remnant. If all cystic duct stumps were shorter than 1.5 cm, would there not be more bile duct injuries? Many cystic ducts either 1) run parallel to the common duct, 2) share a common wall with the common duct, 3) encircle the duct entering on the left side, 4) enter the right hepatic duct, 5) join the common duct very low within the pancreas, or 6) are in close association with the right hepatic artery. Oftentimes, leaving the cystic duct somewhat long, but free of stones, is a reasonable compromise. The report by Rogy *et al.* supports this view suggesting that long-term consequences of a long cystic duct remnant are minimal.

The authors report that all of their patients with long cystic duct stumps presented with pain. They do not comment on the incidence of cholangitis, jaundice, or pancreatitis among a group of patients with common bile duct stones, sphincter of Oddi stenosis and chronic pancreatitis. Certainly, some of their 35 patients must have had these other symptoms. A comparison of the presentation of the 35 patients with a long cystic duct stump and the 245 with "recurrence of benign extrahepatic biliary disease" might also have been enlightening.

Rogy and his associates suggest that endoscopic retrograde cholangiopancreatography (ERCP) should be employed in the workup of these patients. They do not comment, however, on the need for reexploration in their patients with common duct stones when endoscopic sphincterotomy might have been sufficient. Similarly, sphincter of Oddi stenosis can often be diagnosed preoperatively with ERCP delayed emptying, endoscopic manometry, radionuclide common duct-to-duodenum emptying, or ultrasound measured, meal- or choecystokinin-stimulated common duct diameter. Endoscopic sphincterotomy could then be employed without the need for reexploration. Moreover, the authors do not explain why the patients with chronic pancreatitis or hepatitis were explored and what was done to relieve their pain. Finally, no information is provided to document how and for how long these patients were followed and what really happened to their symptoms. Were all of the patients with chronic pancreatitis and sphincter of Oddi stenosis cured by surgery?

Others have reported that excision of a cystic duct stump sometimes relieves pain because of a neuroma in the remnant. Pathologic data on the 35 excised remnants would have been helpful to determine the incidence and significance of neuroma formation. The authors do report that a suture granuloma was the cause of pain in one patient. Was this pathology diagnosed preoperatively? Similarly, was the cyst duct-duodenal fistula diagnosed preoperatively? If these problems were not appreciated by ERCP, as they may not have been, then the yield of reexploration for a cystic duct remnant without preoperatively diagnosed associated pathology might be one or two in the three reported cases.

With the growing enthusiasm for laparoscopic cholecystectomy this report has added significance. The incidence of bile duct injury during laparoscopic cholecystectomy may be as high as 1% to 2%. One recommendation to avoid this problem is to divide the cystic duct as it joins the gallbladder thus leaving a long cystic duct remnant. The report by Rogy and colleagues suggests that this policy is reasonable as long as stones are not left in the remnant. Their data also imply that patients with postcholecystectomy symptoms and a long stone-free duct stump are unlikely to be helped by excision.

Henry A. Pitt
Department of Surgery
John Hopkins University
Baltimore
Maryland, USA

